# Tuning the Properties of Nanogel Surfaces by Grafting Charged Alkylamine Brushes

**DOI:** 10.3390/nano9111514

**Published:** 2019-10-24

**Authors:** Zbyšek Posel, Paola Posocco

**Affiliations:** 1 Department of Informatics, Faculty of Science, Jan Evangelista Purkyně University in Ústí nad Labem, 40096 Ústí nad Labem, Czech Republic; 2 Department of Engineering and Architecture, University of Trieste, 34127 Trieste, Italy; paola.posocco@dia.units.it

**Keywords:** nanogels, charged brushes, dissipative particle dynamics, adsorption, smart surfaces, responsive surfaces

## Abstract

Nanogels are chemically crosslinked polymeric nanoparticles endowed with high encapsulation ability, tunable size, ease of preparation, and responsiveness to external stimuli. The presence of specific functional groups on their surfaces provides an opportunity to tune their surface properties and direct their behavior. In this work, we used mesoscale modeling to describe conformational and mechanical properties of nanogel surfaces formed by crosslinked polyethylene glycol and polyethyleneimine, and grafted by charged alkylamine brushes of different lengths. Simulations show that both number of chains per area and chain length can be used to tune the properties of the coating. Properly selecting these two parameters allows switching from a hydrated, responsive coating to a dried, highly charged layer. The results also suggest that the scaling behavior of alkylamine brushes, e.g., the transition from a mushroom to semi-dilute brush, is only weakly coupled with the shielding ability of the coating and much more with its compressibility.

## 1. Introduction

Polymer brushes are polymer chains covalently linked to a surface. Depending on their grafting density and molecular weight, they might adopt different conformations, which are known to produce mechano-chemical effects, influence the location of the brush terminal groups, and affect access by solvent or other macromolecules to the brush [1,2]. Thus, control of wetting properties, prevention of non-specific binding of biomolecules, colloidal stabilization, and resistance to fouling are all successful examples of polymer brush applications [3].

Synthetic polymer brushes have sparked interest as ‘smart’ interfaces between materials and biological environments in drug delivery and biosensing, and as surfaces for cell-growth and bio-separation [4,5,6,7,8,9,10,11]. Polymer brushes containing stimuli-responsive polymers are ideal building blocks for surfaces that change their macroscopic properties in response to external stimuli [12]. Solvent selectivity, the pH of a surrounding medium, the magnitude of electrostatic forces, may all switch on and off the interaction between a brush and the surroundings. In this context, typical examples are polyelectrolyte brushes. Charged brushes have distinct properties compared to neutral brushes, since the additional interactions due to the charges increase the complexity of the brush structure [13,14].

Surface functionalization with moieties featuring amine groups has been proposed as a strategy to tune the cell adhesion of either cancer or endothelial cells, and to regulate the adsorption of biomolecules from serum-containing medium [15,16,17]. In other works, tertiary and quaternary amino groups were used in the nanogel core to improve the gene delivery within the cytosol [18,19].

In this work, we used mesoscale modeling to describe conformational and mechanical properties of nanogel surfaces [20,21,22,23] formed by crosslinked polyethylene glycol (PEG) and linear polyethyleneimine (PEI). Such PEG/PEI nanoparticles were recently synthetized and tested for drug or gene delivery [24]. They are biodegradable and exhibit good stability and biocompatibility and improved drug release with respect to the bulk gel system. Moreover, the labelling with rhodamine makes them suitable as cell markers. The presence of amino groups on the PEI chain allows for surface functionalization (e.g., by brushes), and this could be used as a strategy to improve and tailor the response of the system to external variables and/or to presence of specific cells in a controlled way. However, the final outcome strictly depends on brush chemistry and level of functionalization, which is hard to predict a priori [25,26].

Thus, this in silico study aims to describe the effect of PEG/PEI nanogel surface functionalization by grafting charged alkylamine brushes of different lengths. The influences of both grafting density σ (e.g., number of chains per nm^2^) and chain length N are taken into account, assuming the coating in presence of water containing a physiological concentration of dissolved salts (NaCl). In addition to environmental conditions, such as temperature or pH, those two parameters (*σ* and *N*) may also contribute to targeting a specific response of the nanogel to the surrounding medium.

The rest of the paper is organized as follows. In Section 2, coarse-grained models of the alkylamine brush, solvent, and PEG/PEI surface are described. Then, we briefly introduce the dissipative particle dynamics simulation method, together with simulation details and observables. In Section 3 we present and discuss major results. First, the swelling behavior of the brush is presented and compared with theory. Then, we analyze the distribution of polymer, solvent, and ions within the polymer layer and correlate it with its ability to respond to the external environment. Afterwards, we describe the mechanical behavior of the coating in terms of osmotic compressibility of the brush. Section 4 then summarizes the results and contains the final discussion.

## 2. Coarse-Grained Modelling

Coarse-graining (CG) is a modelling approach commonly used to reduce the computational cost of complex systems by reducing “unimportant” degrees of freedom. These are then returned back to the system in a more computationally efficient way; for example, as analytical force expression [27]. This makes it possible to simulate materials on large length scales and long time scales. Thus, in a CG model a system is no longer described by atoms or molecules, but by entities called beads. Based on the level of coarse-graining, each bead can represent a collection of atoms or molecules, or larger portions of the real material. Interactions between beads are usually described by potential, soft in nature, as they can in principle partially or completely overlap.

### 2.1. Dissipative Particle Dynamics

Dissipative particle dynamics (DPD) is a well-established CG method that has been successfully used for modeling of complex systems such as polymer brushes, the assembly of copolymers in bulk and in confinement, and the self-assembly of polymer/nanoparticle composites or systems under non-equilibrium conditions, just to mention a few [28,29,30,31,32,33]. Therefore, for details about the method we kindly refer the reader to [34] or [35]. Here, we provide only the details of DPD relevant to our work.

DPD resembles molecular dynamics calculations where the motion of each bead is controlled by a total force $F_{i}$ composed of three pairwise additive forces (Equation (1)):

(1) $$F_{i}=\underset{i,j}{\sum }f_{ij}^{C}\left(r_{ij},a_{ij}\right)+\underset{i,j}{\sum }f_{ij}^{D}\left(\gamma_{ij},r_{ij},v_{ij}\right)+\underset{i,j}{\sum }f_{ij}^{R}\left(\sigma_{ij},r_{ij},\xi_{ij}\right)+f_{i,j}^{b}\left(r_{ij},r_{0},K_{b}\right)+f_{i,j,k}^{\theta }\left(r_{i,j,k},\theta_{0},K_{\theta }\right)$$

These are conservative forces, $f_{ij}^{C}\left(r_{ij},a_{ij}\right)$, dissipative forces, $f_{ij}^{D}\left(\gamma_{ij},r_{ij},v_{ij}\right)$, and random forces,$\;f_{ij}^{R}\left(\sigma_{ij},r_{ij},\xi_{ij}\right)$, where $r_{ij}=r_{i}-r_{j}$, $v_{ij}=v_{i}-v_{j}$, $\xi_{ij}$ is a random number with zero mean and unit variance chosen for each pair (*i*, *j*) of particles independently and $\gamma_{ij}$ and $\sigma_{ij}$ controls the amplitudes of the dissipative and random forces, respectively. Moreover, a cutoff distance $r_{c}$ is included in all the three forces.

For modelling chains, additional contributions are included to describe interactions within the chain. Adjacent beads are connected by bonds described here by an harmonic bond force,$\;f_{i,j}^{b}\left(r_{ij},r_{0},K_{b}\right)$, where $r_{0}$ is the equilibrium distance of the bond and $K_{b}$ is its stiffness. Moreover, the rigidity of the chain is modeled by a bending force, $f_{i,j,k}^{\theta }\left(r_{i,j,k},\theta_{0},K_{\theta }\right)$, where $\theta_{0}$ is the equilibrium angle, $K_{\theta }$ is the stiffness parameter and $r_{i,j,k}$ stands for the distance between i and j beads and between j and k beads, respectively.

In our DPD model, we used standard reduced units where all beads possessed identical mass *m_i_*; the length scale of the system is governed by the cutoff distance $r_{c}$ and the energy is scaled to $k_{B}T$, where $k_{B}$ is the Boltzmann constant and T is the thermodynamic temperature.

### 2.2. Coarse-Grained Models

**Figure 1:**
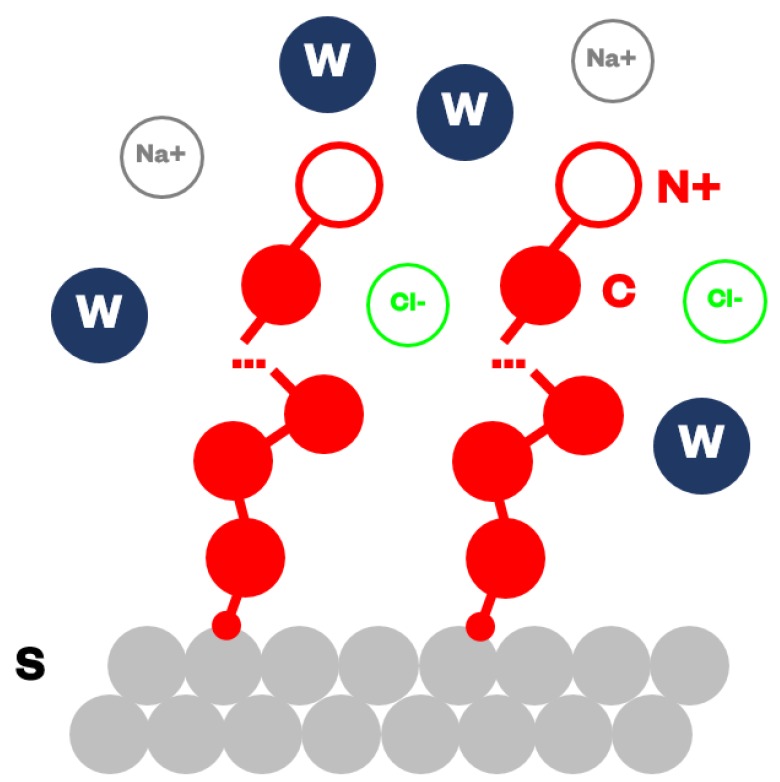
Coarse-grained representation of water, grafted alkylamines, PEG/PEI surface, and Na^+^ and Cl^−^ ions.

To obtain the CG representation of alkylamine brushes in water, shown in Figure 1, we adopted a “top-down” approach based on the systematic parametrization of the water-octanol partition coefficients, as reported in [36]. The level of CG is determined by the number of water molecules in one water bead (W), $N_{m},$ which we set to $N_{m}=2$. Considering the standard DPD relation $\rho r_{c}^{3}=3.0$ and equation $\rho N_{m}v_{m}=1$ [36], we obtained the cutoff distance $r_{c}=0.564\;\mathrm{nm}$ and the weight of one bead as $m_{i}=0.03604\;\mathrm{kg}/\mathrm{mol}$.

Each chain was thus partitioned into beads (connected by bonds) where each bead C contained a CH_2_–CH_2_ group and each terminal bead N+ contained a CH_2_–NH_3_+ group with force parameters $r_{0}=0.39\;r_{c}$, $K_{b}=150\;k_{B}T$, $\theta_{0}=180{}^{\circ}$, and $K_{\theta }=5\;k_{B}T$.

All terminal beads N+ bear a +1 charge, mimicking typical alkylamine protonation state under physiological conditions. In addition to water beads, the solvent contains free ions, Na+ and Cl^−^, with their corresponding ±1 charges, to neutralize the charged brushes and account for ionic strength (C=150 mM. Ions were modeled in partially hydrated forms as single beads, where each Na+ or Cl^−^ bead contained two water molecules as well [37].

Finally, the small size of the grafted molecules relative to the nanogel particle (~200 nm in diameter) allowed it to be modeled as a flat area. The PEG/PEI surface was represented by a collection of “frozen” beads S; i.e. not moving throughout the simulation, and placed parallel to the z direction with thickness equal to $3.0r_{c}.$ The bead density in the surface layer is equal to the density of the fluid; i.e.,$\;\rho r_{c}^{3}=3.0$, to avoid unrealistic density fluctuation of fluid close to the surface. Furthermore, to avoid unphysical penetration of beads through the surface (due to the soft nature of the DPD forces) we placed a reflective layer with bounce-back boundary conditions at the interface between the nanogel surface and the fluid [38]. Each surface bead S was built assuming the same relative ratio of PEG and PEI component used in the synthetic preparation developed by Mauri et al. [24].

Table 1 summarizes the maximum repulsion parameters $a_{ij}r_{c}/k_{B}T$ and corresponding scaled cutoff distances $r_{cs}$ for all the beads calculated, as in [36]. The interaction between the PEG/PEI surface and other components was obtained by weighting pure PEG and PEI interactions where weights were equal to those typically used in the synthetic preparation [24].

Since the DPD forces are soft in nature, the point charges of N^+^, Na^+^ and Cl^−^ groups were replaced by a Gaussian charge distribution [39] to avoid singularities in simulations. For evaluating the electrostatic forces, we employed the standard Ewald summation [40] with identical width of distribution $\sigma =0.8\;r_{c}$ for all charged beads. This allowed us to set the real space cutoff to zero [37] and evaluate only the k-space contribution, where we limited the reciprocal vectors to $k_{max}=\left(5,5,5\right)$. Moreover, we used the homogenous relative dielectric constant of water $\varepsilon_{r}=80$.

In our simulations, we varied the brush molecular weight from ~56 Da to 560 Da, and this led us to N={2, 4, 8, 12, 16, 20} DPD beads per chain. For each chain length N, we systematically varied the grafting density σ={0.4, 0.8, 1.2, 2.5, 5, 6, 7} chains/nm^2^. The total number of grafted chains then increased from 50 for σ=0.4 chains/nm^2^ to 890 for σ=7 chains/nm^2^.

### 2.3. Simulation Details and Observables

All simulations were carried out in LAMMPS package [41] with simulation time step Δt=2.14 ps. Initial configurations and post-processing of equilibrium trajectories were carried out by in-house developed code. First, initial configurations were generated, randomly grafting the chains onto the surface and solvating the resulting system. Then, the structures were equilibrated for $N_{eq}=10^{5}$ simulation steps, e.g., 200 ns, to reach the equilibrium state. The length of the equilibrium period was retrieved from the correlation function of the chain radius of gyration $R_{g}$ calculated for the system with longest chain, n=20, and highest grafting density, σ=7  chains/nm^2^, where the longest correlations were expected. The equilibration period was followed by a production run of $N_{prod}=5\cdot 10^{5}$ steps, e.g., 1 μs, where 5000 uncorrelated frames were collected to calculate the observables. Simulation box dimensions were $L_{x}=L_{y}=\;11.3\;nm$ and $L_{z}=\;26\;nm$. Periodic boundary conditions were applied only in x and y directions, while in z direction we imposed bounce-back boundary conditions.

To characterize the scaling behavior of the polymer brushes, we first calculated the chain mean-squared radius of gyration,$\;R_{g}^{2}$, and obtained the height of the brush as $H=\;R_{gz}$ [42], where *R_gz_* is the component of *R_g_* in the direction *z* perpendicular to the surface.

Averaged density distributions of individual components,$\;\rho_{i}\left(r\right)$, from the nanogel surface to the bulk solvent were measured, where i stands for Na^+^, Cl^−^, water, and chain beads, and they were weighted by the average density of specie i in the system. We also calculated the distribution of charged brush ends and denoted it as $\varphi_{end}\left(r\right)$.

The mechanical response of the brush layer was measured by means of its brush osmotic compressibility [43] as

(2) $$\kappa =\frac{\langle N_{m}^{2}\rangle -\langle N_{m}\rangle {}^{2}}{\langle N_{m}\rangle }\frac{V}{k_{B}T},$$

where $\langle N_{m}\rangle$ is the mean number of chain beads in the element of the volume given by $\left(L_{x}r_{c},\;L_{y}r_{c},0.56r_{c},\right)$ placed in the center of mass of each chain, V is the volume of the simulation box, $k_{B}$ is the Boltzmann constant, T is the thermodynamic temperature, and 〈 〉 denotes ensemble average.

## 3. Results and Discussion

### 3.1. Scaling Behavior of Alkylamine Brushes

Properties of surfaces functionalized by grafted polymers are expected to be governed by the conformation of the polymer chains [9,10,11]. When these are short and the number of grafting points is low, they do not interact with each other, and form isolated islands on the surface. This is known as a “mushroom” regime. Theory predicts that under this condition the brush height *H* scales with the chain length *N* as $H\left(N\right)\propto N^{3/5}$. Increasing the grafting density σ above a crossover value $\sigma_{cross}$, adjacent chains start to overlap, thus inducing steric hindrance and excluded volume effects that eventually result in extended and stretched conformations perpendicular to the surface. That is called the “semi-dilute polymer brush” (SDPB) regime. In that regime, the polymer coating is more compact and in theory the brush height scales as $H\left(N,\sigma \right)\propto \sigma^{1/3}N^{1}$. The conformational transition between mushroom and SDPB occurs at $\sigma_{cross}=\;1/R_{g}^{2}$. At very high grafting densities, the “concentrated polymer brush (CPB)” regime is reached. Here, high-order interactions dominate, and the chains are extended outward from the surface. In CPB, regime *H* scales as $H\left(N,\sigma \right)\propto \sigma^{1/2}N^{1}$.

In contrast, the chain conformation of polyelectrolyte brushes is mainly determined by electrostatic interactions between charged monomers and by the osmotic pressure of counterions. Different regimes may exist depending on the chain length, grafting density, charge fraction, and ionic strength of the solution (e.g., osmotic, salted, and Pincus brushes) [44]. However, weakly charged brushes may behave like neutral brushes when the amount of charged monomers is not sufficient to overcome excluded volume interactions and entropic stretching [45].

**Figure 2:**
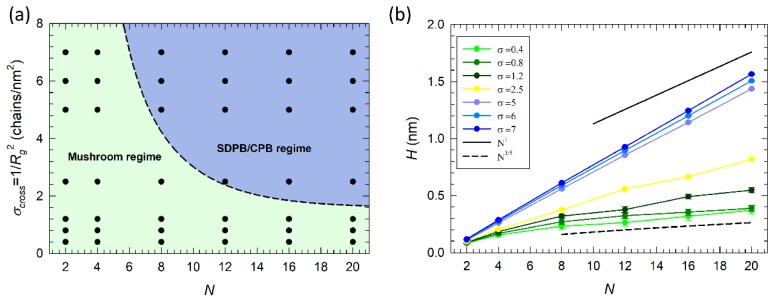
Scaling behavior of alkyl amine brushes grafted to a PEG/PEI surface. (**a**) Crossover grafting density $\sigma_{cross}$ from mushroom (green filled area) to semi-dilute polymer brush (SDPB) regime (blue filled area) as a function of the grafted chain length N. Black points denote systems considered in this study. (**b**) Scaling behavior of the brush height *H* as a function of N for all grafting densities considered in this study. A black solid line indicates scaling of the brush in SDPB regime, as $H\;\left(N,\sigma \right)\propto N^{1}$, and a black dashed line indicates scaling in mushroom regime, $H\left(N\right)\propto N^{3/5}$.

Thus, we first performed a series of simulations of free isolated chains in solution, calculated the chain squared radius of gyration, $R_{g}^{2}$, and in turn the crossover grafting density, $\sigma_{cross}.$ Figure 2a displays all different coatings considered in this study coupled with the theoretical $\sigma_{cross}$ (dashed line). Coatings in the green area belong to the mushroom regime, while coatings in blue area have brushes in SDPB/CPB regimes. Short chains, N={2, 4} or sparsely grafted chains, σ≤1.2 chains/nm^2^, are fitting in the mushroom regime, while an SDPB/CPB state is achieved with densely grafted, σ≥2.5 chains/nm^2^ and longer chains, N>8.

This conformational transition is also reflected in different scaling behavior of the brush height, H(N), as a function of brush length N (Figure 2b). Dashed and solid black lines in the figure denote theoretical mushroom (dashed line) and SPBD scaling (solid line), respectively. Both scaling regimes were observed. First, the mushroom regime, σ∈(0.4, 1,2) chains/nm^2^, where *H* is almost independent from σ and the scaling exponent is close to 3/5. Then, the transition between mushroom and SDPB regime takes place at =2.5 chains/nm^2^. Finally, systems with σ>2.5 chains/nm^2^ have scaling close to an SDPB regime where the deviation from the ideal exponent 1 can be addressed to σ scaling exponent. The data suggest that the swelling behavior of *H* for these cationic brushes is close to that of neutral ones, likely due to the relatively small fraction of charged units. Thus, chain-chain/chain-solvent interactions as well as entropic elasticity dominate and overall determine the brush behavior.

### 3.2. Distribution of Polymer, Water, and Ions

**Figure 3:**
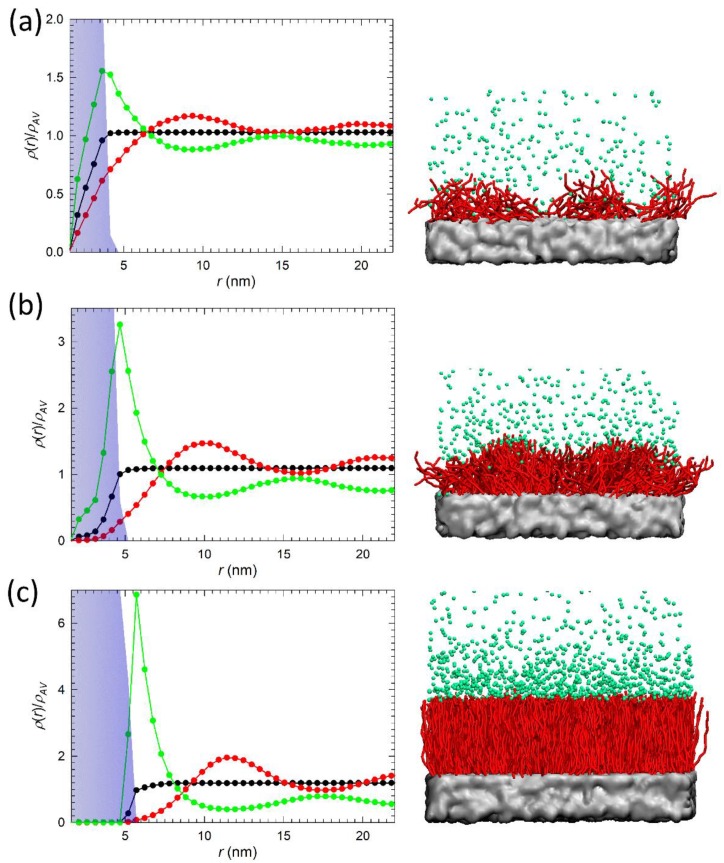
Left column: Averaged density distribution ρ(r) for coatings grafted by alkylamine chains with N=12 beads at (**a**) low grafting density $\sigma =\;0.8\;chains/nm^{2}$, (**b**) intermediate grafting density $\sigma =\;2.5\;chains/nm^{2}$, and (**c**) high grafting density $\sigma =\;6\;chains/nm^{2}$. Each density distribution is scaled to the average density of the species, $\rho_{AV}$, in the system. Green, red, and black lines represent anions, cations, and water, respectively. The blue area highlights the coating layer. Right panel: corresponding simulation snapshots where cations and water beads are omitted for clarity. Green spheres represent anions, red sticks the alkylamine chains, and the PEG/PEI surface is displayed in silver.

Figure 3 reports the density distribution ρ(r) for each species (polymer, water, and ions) at distance *r* measured from the PEG/PEI surface for selected system. The blue filled area represents the alkylamine coating, while the green, red, and black lines refer to anions, cations, and water distributions, respectively. The values are scaled by their average density, $\rho_{AV}.$ Right panels in Figure 3 show the corresponding simulation snapshot as a front view of the system, where Na+ and water beads were omitted for clarity. Here, we present the results only for an intermediate chain length N=12. Additional systems are shown in Figures S1 and S2 in Supporting Information.

**Figure 4:**
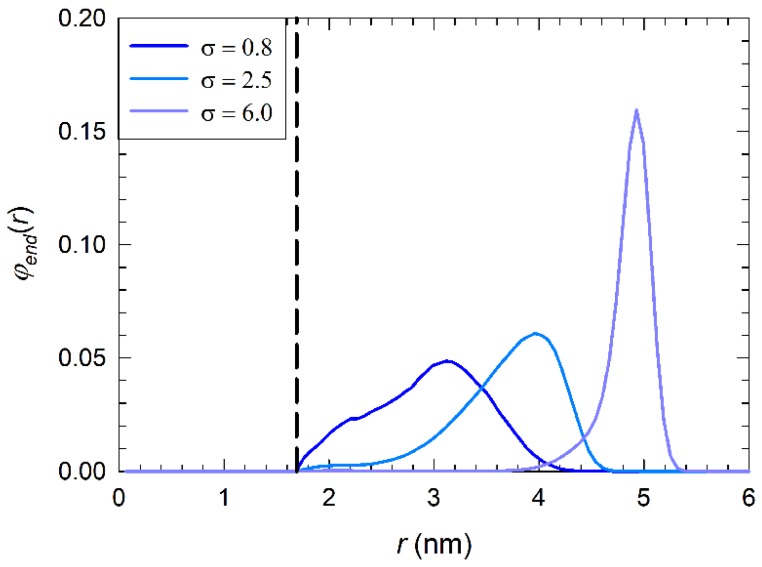
End monomer distribution, $\varphi_{end}\left(r\right)$, of alkylamine chains with N=12 shown only for selected grafting densities σ. The black dashed line indicates the position of PEG/PEI surface. A complete set of end-monomer distributions is reported in Supporting Information in Figure S3.

At low grafting density, $\sigma =\;0.8\;chains/nm^{2},$ (Figure 3a), water and ions may permeate the polymeric layer. The maximum observed in the density of Cl^−^ distribution at the coating/water interface suggests that anions in solution concentrate rather close to the solvent/coating interface to compensate the positive charge of the alkylamine chains. The corresponding distribution of charged end-monomers, $\varphi_{end}\left(r\right)$, is shown in Figure 4 for comparison. Thus, when the chains are in mushroom regime, a good level of hydration is observed with sustained diffusion of ions within the layer.

Increasing the grafting density to $\sigma =\;2.5\;chains/nm^{2}$ brings the chains close to the transition from mushroom to SDPB/CPB regime (see Figure 2a) and part of the chains adopt a more stretched conformation due to the onset of chain-chain excluded volume interactions (see right panel in Figure 3b). Therefore, positively charged trimethylamines are confined closer to the water/coating interface (see Figure 4) and promote the layering of anions on the top of the coating. This is evidenced also by an almost doubled concentration of Cl^−^ residing at the interface with respect to low *σ* values. The increased chain confinement and Cl^−^ layering leads to an overall decrease of Na+ and water molecules inside the coating. Cations are mostly repelled from the coating to the bulk solvent. Water diffusion within the polymer layer is hindered by the increased density of the hydrophobic tails of the brushes inside the coating.

Finally, at high grafting density, $\sigma =\;6\;chains/nm^{2}$, (Figure 3c) all chains are well into the SDPB/CPB regime and adopt a stretched conformation oriented perpendicularly to the surface. As a consequence, the positive charge of the brush is almost exclusively located at the solvent/coating interface (see the narrow distribution of end-monomers in Figure 4) and promotes the formation of a dense negatively charged external layer (seen also in the right panel in Figure 3c), evidenced by the enhanced peak in Cl^−^ density distribution. The high positive surface charge, together with the presence of a dense layer of anions on the top of the coating and the increased density of the hydrophobic chain tails leads to shielding of PEG/PEI surface from the surrounding environment and the scaled density of water and ions drops to zero close to the solvent/coating interface.

**Figure 5:**
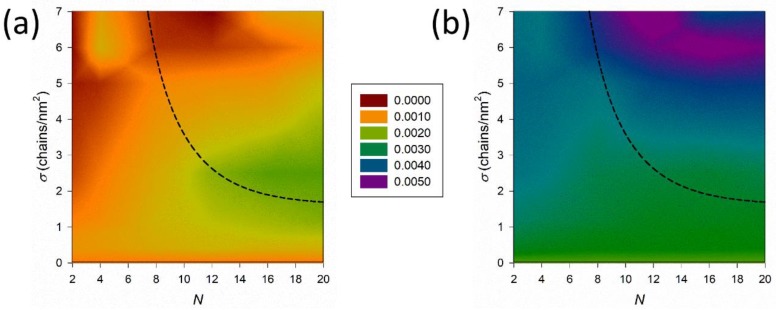
2D density maps of (**a**) Na^+^ and (**b**) Cl^−^ calculated at water/coating interface and displayed by color-scale in N-σ plane. The black dashed line indicates the brush transition from mushroom to SDPB regime.

Overall, the evidence seems to suggest a correlation between the brush regime and the hydration behavior of the alkylamine brushes. However, a more complex picture could be retrieved by comparing the organization of ions at the solvent/coating interface summarized in Figure 5, in terms of 2D density maps for all systems. The water density distribution is not reported here since it is similar for all systems and close to that of bulk water. The solvent/coating interface represents the outer boundary exposed to the external environment and was calculated at the distance where the polymer average density decreased to zero. The densities of cations (Figure 5a) and anions (Figure 5b), are displayed by color-scale in N−σ plane. Values at $\sigma =\;0\;chains/nm^{2}$ refers to a surface not grafted by any alkylamine chain. Dashed black line shows the crossover grafting density $\sigma_{cross}$ discussed in Figure 2a.

Regardless the brush length *N*, at grafting density $\sigma >\;4\;chains/nm^{2}$ the interface presents a dense, negatively charged layer induced by the surface confinement of the brush end groups (see corresponding end-monomer distributions in Figure S3c and snapshots of highly grafted surface in Figure 3c, Figures S1c and S2c). This also restricts the access of cations to the coating and their interfacial density is almost zero. The behavior does not depend on the brush conformation state (mushroom versus SDPB/CPB).

Decreasing the grafting density to $\sigma <\;2.5\;chains/nm^{2}$ leads to a different organization of the cations at the interface. The brushes in mushroom regime that are limited by short chain lengths, N<12, behave as isolated chains that do not confine the positive charge at the interface, nor may they form a hydrophobic layer that decreases the penetration of ions and water. Therefore, the cations may adopt an organization similar to that of an ungrafted surface. On the contrary, longer alkylamines (N ≥12) adopt a more coiled conformation that provide a denser hydrophobic layer that repels cations closer to the solvent/coating interface, as evidenced by the green area in Figure 5a. The lack of confined positive charge and subsequent anion layering is evidenced by their constants surface density in Figure 5b.

**Figure 6:**
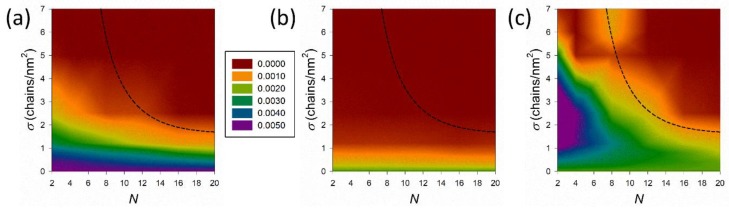
2D density maps of (**a**) water, (**b**) Na^+^, and (**c**) Cl^−^ obtained close to the PEG/PEI surface and displayed by color-scale in N – σ plane. Black dashed line indicates the brush transition from mushroom to SDPB regime.

In addition to the solvent/coating interface, an additional interesting piece of information can be retrieved by analyzing density distributions within the coating layer and close to the PEG/PEI surface. This provides hints of the accessibility of the brush by the solvent. We plotted 2D density maps for water molecules, cations, and anions close to the PEG/PEI surface in Figure 6a–c, respectively.

From Figure 6a, we see that coatings obtained with low grafting density do not screen out the PEG/PEI surface from the external environment, and water and ions may diffuse inside the brush. Increasing the grafting density, $\sigma >2\;chains/nm^{2}$, the hydration level decreases drastically and for higher *σ* the coating is almost dried. Thus, the distribution of water molecules within the polymer layer is mainly dictated by the grafting density *σ* and it does not depend on the chain length *N*. An increase in the grafting density results in a dense and compact hydrophobic layer that avoids water penetration regardless the brush regime.

Distribution of Na+ inside the coating (Figure 6b) is governed mainly by confinement of alkyl amine positive charge close to solvent/coating interface that screen them out, as was previously discussed in Figure 3, Figure 4 and Figure 5b. Therefore, the density of cations drops to zero near the PEG/PEI surface for coatings with $\sigma >\;1\;chains/nm^{2}$.

**Figure 7:**
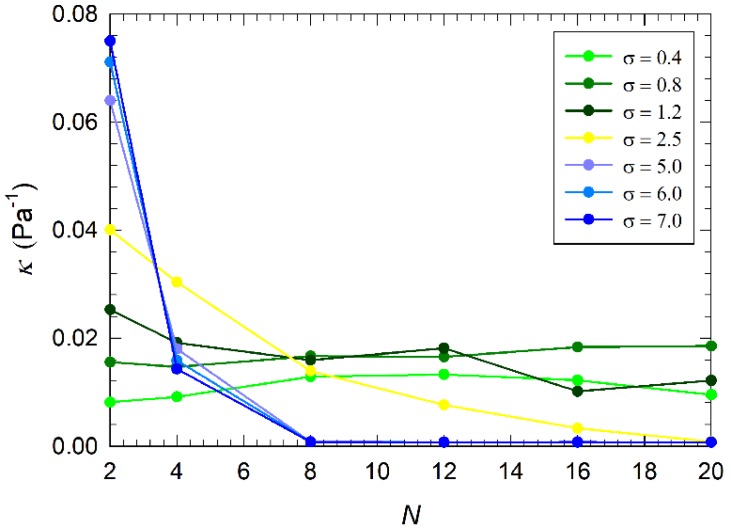
Osmotic compressibility κ as a function of the grafted chain length *N* for all grafting densities *σ* considered in this study.

Finally, the interplay between hydrophobic repulsion and confinement of positively charged amine near the water/coating interface determines the distribution of Cl^−^ in Figure 6c. We see that coatings with $\sigma ≲\;1\;chains/nm^{2}$ are highly permeable regardless the chain length N. Increasing the grafting density leads to the confinement of chain end groups close to solvent/coating interface that induce the formation of an anion layer on the top of the surface. Layered anions tend to penetrate inside the coating increasing its osmotic compressibility (see Figure 7 and discussion below). This pressure increases with increasing grafting density σ and chain length N. Nevertheless, long chains in the coating effectively fill the space among grafted chains and form a compact hydrophobic layer that resists the pressure of external anions and prevents their diffusion to the PEG/PEI surface. Despite the coating composed of short chains also forming a hydrophobic layer, its compactness is lower and cannot resist the pressure of layered anions. We see that shorter the grafted chain is, the higher the grafting density must be to resist the permeation of anions to PEG/PEI surface.

### 3.3. Osmotic Compressibility of the Coating

In addition to chain conformation and ability of the coating to be impermeable to ions and water, another fundamental feature for modified surfaces is their mechanical response to external stimuli. Here we analyze it by plotting the osmotic compressibility κ of the alkylamine brush as a function of chain length N (Figure 7). In addition to excluded volume effects and entropy elasticity that control the properties of coatings composed of neutral brushes, electrostatic interactions become important in charged brushes. The presence of charged particles inside the coating may increase its osmotic pressure, i.e., osmotic compressibility, and the tendency to be swollen by the surrounding solvent.

As discussed earlier, the diffusion of ions Cl^−^ into the coating is a result of interplay between pressure of anions layering on the top of the coating and the formation of a compact hydrophobic layer close to PEG/PEI surface that prevents it. Therefore, we measured the osmotic compressibility inside the coating at position of center-of-mass of each chain; e.g., in between solvent/coating interface and PEG/PEI surface (distribution of ions and water shown in Figure 5 and Figure 6).

Coatings with low grafting density, $\sigma \leq \;1\;chains/nm^{2}$, may be permeated by ions and water molecules regardless the grafted chain length, which leads to comparable osmotic compressibilities. Increasing the grafting density to $\sigma =\;2.5\;chains/nm^{2}$ induces layering of anions on the top of the coating and their enhanced pressure leads to an increased permeation of anions into the coating composed of short chains, as depicted by an increased osmotic compressibility (yellow line in Figure 7). Nevertheless, increasing the chain length leads to the formation of a more compact hydrophobic layer that prevents permeation of anions, thus decreasing the osmotic compressibility. A similar trend was also observed for coatings with higher grafting density. A sharp transition is observed at chain length N=8 where the osmotic compressibility drops to zero.

## 4. Conclusions

In this work, we applied the dissipative particle dynamics simulation method to describe conformational and mechanical properties of nanogel nanoparticles formed by crosslinked polyethylene glycol and polyethyleneimine and grafted by charged alkylamine brushes. We systematically varied the brush chain length and grafting density to describe the relation between brush conformation and properties of the coating, including its mechanical response, and the ability to shield or be permeated by the surrounding solvent. For that, coarse-grained models of the nanogel nanoparticle, alkylamine chains, and solvent were constructed.

First, we compared the scaling behavior, in terms of alkylamine brush height, with the scaling theory. We showed that charged alkylamine brushes follow the same scaling behavior of neutral brushes rather than that predicted for polyelectrolyte brushes.

Scaled density distributions were then used to detail the behavior at the solvent/coating interface and close to the PEG/PEI surface. Surfaces sparsely grafted by short chains exhibit a good level of hydration, as ions and water may permeate through the coating to the PEG/PEI surface. Increasing the chain length led to the formation of a hydrophobic layer close to the PEG/PEI surface that partially repelled cations closer to the solvent interface. For these coatings the chains were in mushroom regime and no confinement of their terminal groups and subsequent layering of anions on the top of the coating was observed. Increasing the grafting density brought the chains to the transition between mushroom and semi-dilute polymer brush regime. Here, we observed a confinement of alkylamine positive charges closer to the interface, which in turn induced the formation of anion layer on the top of the coating and of a hydrophobic layer close to PEG/PEI surface. This resulted in a decreased level of surface hydration. Further increase of the grafting density emphasized the compactness of the hydrophobic layer and the layering of anions, which prevented the permeation of ions from the bulk to the interface.

Finally, we showed that the mechanical responses of alkylamine coatings, e.g., their osmotic compressibility, correlate with their ability to be permeated or shield ions and water, and that both can be tuned by properly selecting the chain length and grafting density.

We think that this study provides a helpful tool to design PEG/PEI-based nanogels with desired interfacial properties. We also believe that the results presented here offer insightful molecular details, which can be of support for those developing new smart surfaces, modified by weakly charged brushes.

## Figures and Tables

**Table 1:** Maximum repulsion parameters $a_{ij}r_{c}/k_{B}T$ and scaled cutoff distances $r_{cs}$ (in the brackets) listed for all the components in the coarse-grained system.

Bead Type	W	C	N^+^	Na^+^	Cl^−^	S
**W**	25.00 (1.000)	45.00 (1.0370)	14.50 (1.0120)	25.0 (1.000)	25.00 (1.000)	24.64 (1.0450)
**C**	-	22.00 (1.0740)	22.50 (1.0490)	45.0 (1.0370)	45.00 (1.0370)	26.34 (1.0820)
**N^+^**	-	-	21.50 (1.0240)	14.50 (1.0120)	14.50 (1.0120)	20.92 (1.0570)
**Na^+^**	-	-	-	25.00 (1.000)	25.00 (1.000)	24.64 (1.0450)
**Cl^−^**	-	-	-	-	25.00 (1.000)	24.64 (1.0450)
**S**	-	-	-	-	-	25.00 (1.000)

## Supplementary Materials

The following are available online at https://www.mdpi.com/2079-4991/9/11/1514/s1, Figure S1: Left column: Averaged density distribution ρ(r) for coatings grafted by alkylamine chains with N=4 beads at (a) low grafting density $\sigma =\;0.8\;chains/nm^{2}$, (b) intermediate grafting density $\sigma =\;2.5\;chains/nm^{2}$ and (c) high grafting density $\sigma =\;6\;chains/nm^{2}$. Each density distribution is scaled to the average density of the specie, $\rho_{AV}$, in the system. Green, red and black lines represent anions, cations and water, respectively. The blue area highlight the coating layer. Right panel: corresponding simulation snapshots where cations and water beads are omitted for clarity. Green spheres represent anions, red sticks the alkylamine chains and the PEG/PEI surface is displayed in silver, Figure S2: Left column: Averaged density distribution ρ(r) for coatings grafted by alkylamine chains with N=20 beads at (a) low grafting density $\sigma =\;0.8\;chains/nm^{2}$, (b) intermediate grafting density $\sigma =\;2.5\;chains/nm^{2}$ and (c) high grafting density $\sigma =\;6\;chains/nm^{2}$. Each density distribution is scaled to the average density of the specie, $\rho_{AV}$, in the system. Green, red and black lines represent anions, cations and water, respectively. The blue area highlight the coating layer. Right panel: corresponding simulation snapshots where cations and water beads are omitted for clarity. Green spheres represent anions, red sticks the alkylamine chains and the PEG/PEI surface is displayed in silver, Figure S3: End monomer distribution, $\varphi_{end}\left(r\right)$, of all alkylamine chains at (a) low grafting density,$\;\sigma =0.8\;chains/nm^{2}$, (b) intermediate grafting density, $\sigma =2.5\;chains/nm^{2}$, and (c) at high grafting density $\sigma =6\;chains/nm^{2}$. Black dashed line indicates the position of PEG/PEI surface.
