# Supplementary material for: Tuning the Properties of Nanogel Surfaces by Grafting Charged Alkylamine Brushes
Source: Nanomaterials (Basel). 2019 Oct 24;9(11):1514. doi: 10.3390/nano9111514 (PMC6915512; doi:10.3390/nano9111514)
Supplement: Supplementary file 1 [file nanomaterials-09-01514-s001.pdf]

nanomaterials
MDPI

SUPPORTING INFORMATION

Tuning the properties of nanogel surfaces by grafting charged alkylamine brushes

Zbyšek Posel[1,2*] and Paola Posocco[2]

[1] Department of Informatics, Faculty of Science, Jan Evangelista Purkyně University in Ústí nad Labem, Czech Republic; zbysek.posel@ujep.cz
[2] Department of Engineering and Architecture, University of Trieste, Italy; paola.posocco@dia.units.it
Correspondence: zbysek.posel@ujep.cz

Abstract: Nanogels are chemically crosslinked polymeric nanoparticles endowed with high encapsulation ability, tunable size, ease of preparation, and responsiveness to external stimuli. The presence of specific functional groups on their surfaces provides an opportunity to tune their surface properties and direct their behavior. In this work, we used mesoscale modeling to describe conformational and mechanical properties of nanogel surfaces formed by crosslinked polyethylene glycol and polyethyleneimine and grafted by charged alkylamine brushes of different length. Simulations show that both number of chains per area and chain length can be used to tune the properties of the coating. Properly selecting these two parameters allows switching from a hydrated, responsive coating to a dried, highly charged layer. The results also suggest that the scaling behavior of alkylamine brushes, e.g. the transition from mushroom to semi-dilute brush, is only weakly coupled with the shielding ability of the coating and much more with its compressibility.

ADDITIONAL RESULTS

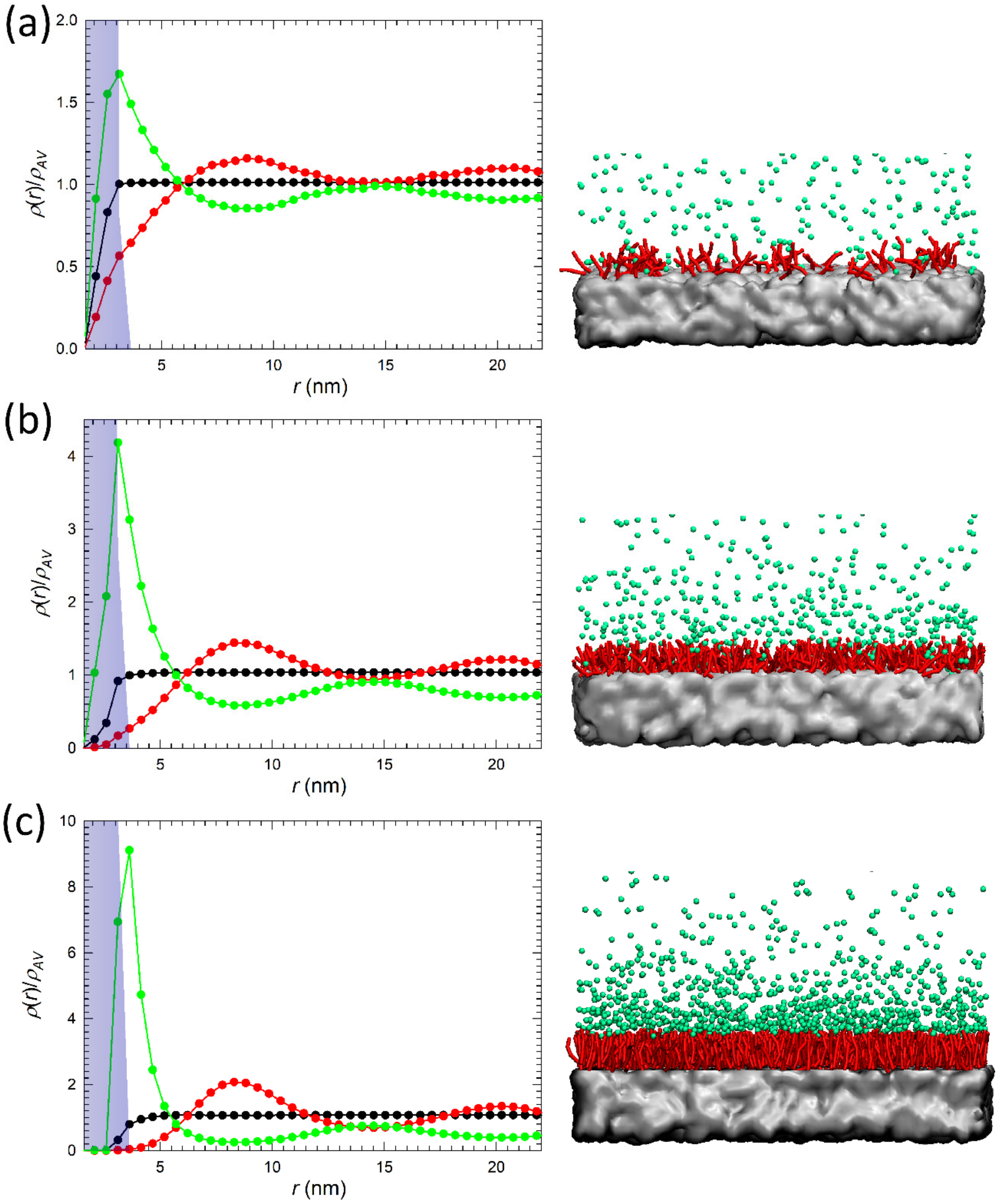

Figure S1. Left column: Averaged density distribution $\rho(r)$ for coatings grafted by alkylamine chains with $N = 4$ beads at (a) low grafting density $\sigma = 0.8\ chains/nm^2$, (b) intermediate grafting density $\sigma = 2.5\ chains/nm^2$ and (c) high grafting density $\sigma = 6\ chains/nm^2$. Each density distribution is scaled to the average density of the specie, ρ_{AV}, in the system. Green, red and black lines represent anions, cations and water, respectively. The blue area highlight the coating layer. Right panel: corresponding simulation snapshots where cations and water beads are omitted for clarity. Green spheres represent anions, red sticks the alkylamine chains and the PEG/PEI surface is displayed in silver.

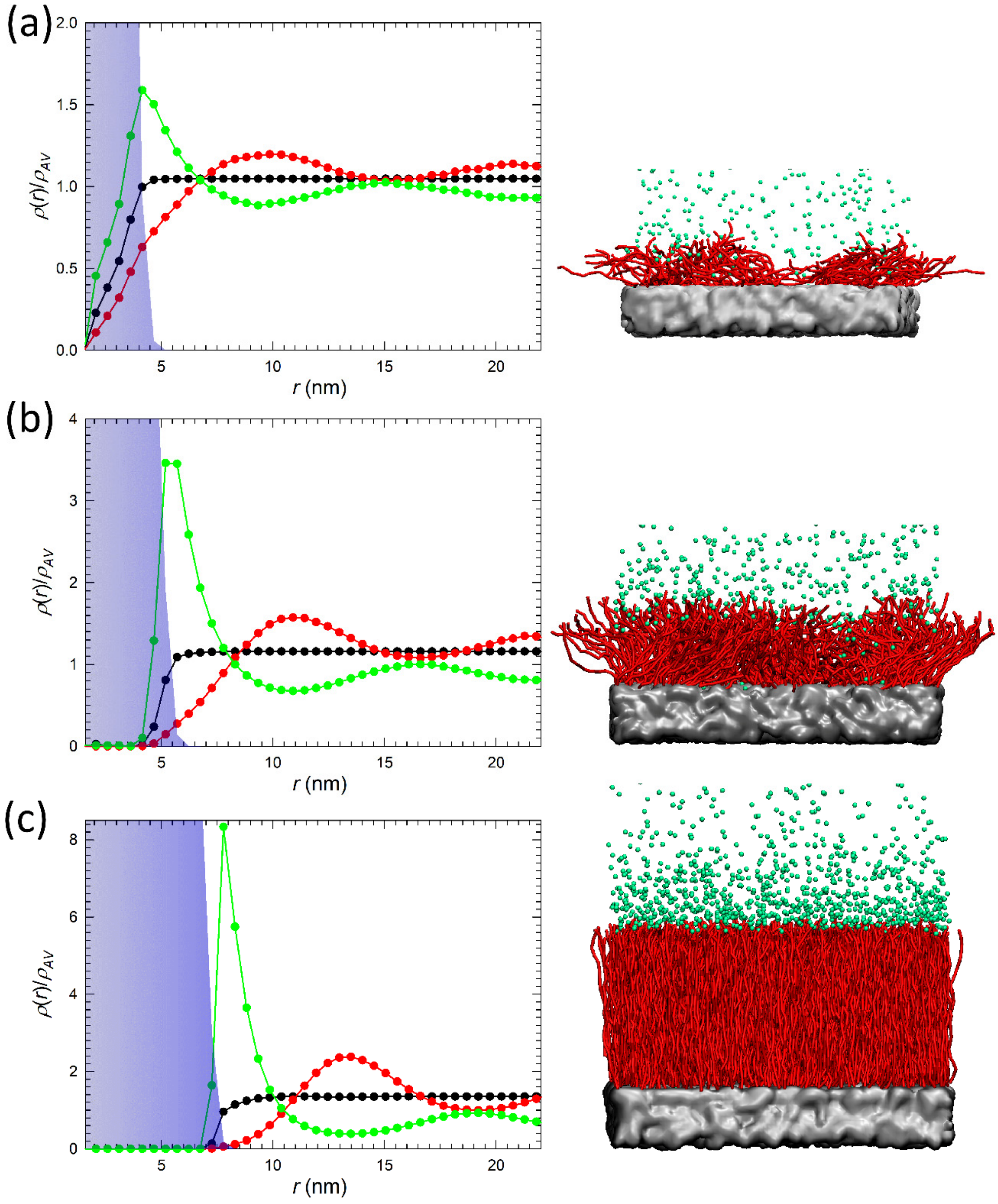

Figure S2. Left column: Averaged density distribution $\rho(r)$ for coatings grafted by alkylamine chains with $N = 20$ beads at (a) low grafting density $\sigma = 0.8\ chains/nm^2$, (b) intermediate grafting density $\sigma = 2.5\ chains/nm^2$ and (c) high grafting density $\sigma = 6\ chains/nm^2$. Each density distribution is scaled to the average density of the specie, ρ_{AV}, in the system. Green, red and black lines represent anions, cations and water, respectively. The blue area highlight the coating layer. Right panel: corresponding simulation snapshots where cations and water beads are omitted for clarity. Green spheres represent anions, red sticks the alkylamine chains and the PEG/PEI surface is displayed in silver.

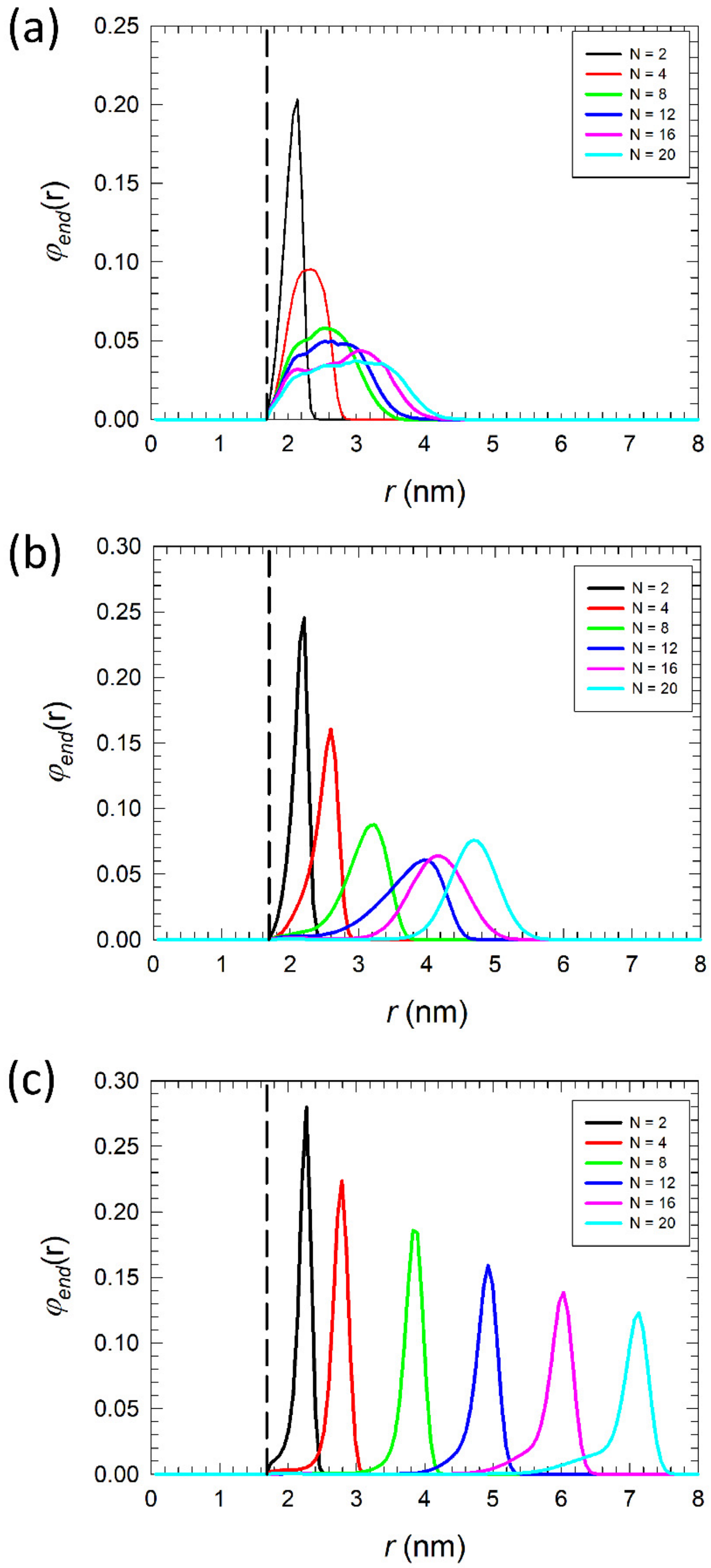

Figure S3. End monomer distribution, $\varphi_{end}(r)$, of all alkylamine chains at (a) low grafting density, $\sigma = 0.8\ chains/nm^2$, (b) intermediate grafting density, $\sigma = 2.5\ chains/nm^2$, and (c) at high grafting density $\sigma = 6\ chains/nm^2$. Black dashed line indicates the position of PEG/PEI surface.
